# ChREBPα: a central metabolic sensor driving lipid droplet renewal in preimplantation mouse embryos

**DOI:** 10.3389/fcell.2026.1793255

**Published:** 2026-04-23

**Authors:** Abdul Majid Khan, Dawid Winiarczyk, Francesca Boffa, Dominika Żbikowska, Luca Palazzese, Silvestre Sampino, Domenico Iuso

**Affiliations:** 1 Department of Experimental Embryology, Institute of Genetics and Animal Biotechnology of the Polish Academy of Sciences, Jastrzębiec, Poland; 2 Department of Veterinary Medicine, University of Teramo, Teramo, Italy

**Keywords:** ACSL, ChREBPα, lipid droplets (LDs), SBI-993, Triacsin C

## Abstract

**Background:**

Lipid droplets (LDs) are abundant during early embryogenesis; however, yet, the mechanisms that govern their synthesis, maintenance, and functional relevance remain poorly defined. This study aimed to investigate how early mouse embryos sense lipid depletion and activate adaptive metabolic responses to sustain developmental progression.

**Methods:**

Mouse zygotes were mechanically delipidated and cultured under fatty acid–free conditions, and LD recovery was quantified by BODIPY staining across development. RNA sequencing was performed at the 2-cell stage embryos to identify transcriptional responses to delipidation. Furthermore, immunofluorescence microscopy, quantitative assays, and functional studies were conducted by the use of inhibitors to investigate molecular actors of LDs *de novo* synthesis and their role in embryonic survival.

**Results:**

Delipidated embryos rapidly regenerated LDs and progressed to the blastocyst stage at rates comparable to controls. Transcriptomic profiling identified ChREBP (Mlxipl) as the principal gene upregulated in response to lipid depletion, with both mRNA and protein levels markedly increased in delipidated embryos. ChREBP displayed dynamic subcellular localization, including nuclear accumulation associated with LD biogenesis and the formation of cytoplasmic foci preferentially localized at the cell cortex and in proximity to endoplasmic reticulum–enriched regions. Partial co-localization of ChREBP with LDs was observed across stages of *de novo* LD synthesis following delipidation. Furthermore, an association with lipid LDs was observed during active LD regeneration following delipidation. Notably, the inhibition of ChREBP impaired lipid droplet regeneration and resulted in developmental arrest, identifying the morula-to-blastocyst transition as a critical window of sensitivity and the trophectoderm formation as a vulnerable bottleneck.

**Discussion:**

These findings identify ChREBP as a key metabolic sensor that coordinates LD synthesis and supports normal developmental progression during early embryogenesis, highlighting LDs as critical regulators of early embryonic competence.

## 1 Introduction

The regulation of lipid metabolism and glucose homeostasis is critical for maintaining cellular and systemic energy balance during embryonic development. LDs are dynamic intracellular organelles composed of a neutral lipid core surrounded by a phospholipid monolayer and play an essential role in lipid storage and metabolism across various tissues (Olzmann and Carvalho, 2019). In mammalian oocytes and preimplantation embryos, LDs are highly abundant, and their content and distribution vary markedly among the species. Domestic animals such as sheep, bovine, and horses exhibit a substantially higher LDs content in oocytes and early embryos compared with human and mouse, with additional variability also reported among different mouse strains (Ibayashi et al., 2021). For many years, the functional relevance of LDs during early development remained enigmatic, in part because mechanical delipidation of oocytes did not impair development to the blastocyst stage and viable offspring (Arena et al., 2021), leading to the assumption that LDs are largely dispensable. Interesting studies, however, began to challenge this view by demonstrating a role of LDs as an energetic reservoir during blastocyst diapause, supporting embryonic survival under conditions of metabolic dormancy (van der Weijden et al., 2024).

More recently, multiple studies now indicate the essential nature of LD dynamics, synthesis, and maintenance during early development (Aizawa et al., 2019). Chemical inhibition of lipid droplet biogenesis in mouse models results in severe developmental arrest at the morula stage, underscoring their requirement for successful preimplantation development (Tatsumi et al., 2018). In addition, lipid droplet enlargement and mobilization have been shown to coordinate morphogenetic events during peri-implantation development in mice (Mau et al., 2022), further implicating these organelles in developmental patterning and differentiation processes (Watanabe et al., 2010; Li et al., 2023). Notably, LDs are actively synthesized and maintained throughout early embryogenesis and are rapidly reconstituted following mechanical removal, indicating the existence of a robust and adaptive metabolic program capable of restoring lipid storage capacity (Aizawa et al., 2019; Mau et al., 2022). Despite the growing recognition of LDs as essential organelles during early embryogenesis, the transcriptional and metabolic programs that sense nutrient availability and coordinate LD biogenesis remain largely undefined. While nutrient-sensing pathways such as AMPK and mTOR have been extensively studied in somatic cells (González et al., 2020), how metabolic sensing is transcriptionally integrated with lipid metabolism during early embryonic development remains poorly understood (Scott et al., 2018).

In the present study, we identify the carbohydrate-responsive element-binding protein (ChREBPα) as a key transcriptional regulator orchestrating LD biogenesis and lipid metabolic pathways essential for blastocyst development. Our findings establish ChREBP-α as a lipid and energy sensor in early mouse embryos and reveal its central role in mediating an adaptive metabolic response to energy deficiency, which is critical for embryonic survival and developmental competence.

## 2 Materials and methods

### 2.1 Animals and ethics statements

All experiments were conducted in accordance with the Polish Governmental Act for Animal Care and were approved by the local Animal Care Committee of the Warsaw University of Life Sciences (approval no. WAW2/62/2023). Experiments were performed on F1 (C57Bl/10 × CBA/H) mice at the Institute of Genetics and Animal Biotechnology, Polish Academy of Sciences. Animals were housed in 30.5 cm × 13 cm × 11 cm cages in a temperature-controlled room under a 12 h/12 h light/dark cycle (lights on from 06:00 to 18:00). Food (Labofeed H, Kcynia, Poland; metabolizable energy 13.0 MJ/kg) and water were provided *ad libitum*.

### 2.2 Collection of zygotes

Hormonal stimulation was conducted as reported previously (Ziętek and Sampino, 2023; Winiarczyk et al., 2025). Briefly, donor F1 females (2–5 months old) were stimulated for ovulation by intraperitoneal injection of 7.5 IU (0.1 mL) pregnant mare serum gonadotropin (PMSG; Cat# RP17827225000, BioVendor, Czech Republic), followed 48 h later by 7.5 IU (0.1 mL) human chorionic gonadotropin (hCG; Cat# 018533, MSD Animal Health, Netherlands). Females were then mated with F1 stud males. The following morning, females were checked for the presence of a copulatory plug. Females with copulation plugs were transferred to the laboratory, and zygote-stage embryos were harvested from the oviducts under a stereomicroscope. Cumulus cells were removed by incubation in M2 medium (Sigma-Aldrich) containing 0.1% hyaluronidase (Sigma-Aldrich), followed by multiple washes in fresh M2 medium.

### 2.3 Delipidation

Lipids were removed from zygote-stage embryos as previously described (Aizawa et al., 2019). The entire experiment was performed on a pre-warmed plate to maintain physiological conditions. Thirty zygotes were initially placed into a 1.5 mL microcentrifuge tube containing 500 µL M2 medium supplemented with cytochalasin B at a final concentration of 50 µg/mL. A first centrifugation was applied at 4,200 × *g* for 10 min to polarize LDs within the blastomere. After the first centrifugation, 250 µL of pre-warmed hyperosmotic solution (M2 medium supplemented with cytochalasin B and 50 µg/mL sucrose) was added to the microcentrifuge tube. The embryos were then subjected to a second centrifugation at 9,500 × *g* for 10 min to move the LD aggregates into the perivitelline (PV) space. Then, embryos were transferred to fresh M2 medium containing cytochalasin B 50 µg/mL under the micromanipulation chamber. A small opening was created in the zona pellucida using laser-assisted manipulation, after which the biopsy was mechanically performed by aspirating the LD into a pipette with a 12 µm inner diameter. All manipulations were performed under an Inverted microscope Nikon Eclipse Integra Ti (Nikon Corporation, Japan) using an Eppendorf Micromanipulator 5,176 (Eppendorf AG, Germany). Following manipulation, embryos were thoroughly washed in M2 medium and returned to culture in free fatty acid KSOM under standard conditions.

### 2.4 Embryo culture and treatments

Zygotes were cultured in fatty acid-free KSOM previously describe (Behringer et al., 2014) under Mineral oil at 37.5 °C in a humidified atmosphere of 5% CO_2_ and atmospheric O_2_ from 0.5*dpc* to 4.5*dpc* (zygote to blastocyst stage). An experiment was conducted to assess the developmental potential of delipidated zygotes under different culture conditions and to examine LD reconstitution in the absence of specific nutrients. Delipidated zygotes were cultured in four different conditions: 1. complete free fatty acid KSOM, 2. KSOM without pyruvate, 3. KSOM without glucose, and 4. KSOM without glutamine. LDs were monitored at the 2-cell stage to evaluate their reconstruction under each condition.

Next, a part of zygotes were cultured *in vitro* (IVC) in free fatty acid KSOM medium supplemented with Triacsin C, a potent inhibitor of long-chain acyl-CoA synthetases (ACSLs). Triacsin C was dissolved in DMSO and initially tested at concentrations of 5, 12.5, and 25 µM. Based on these preliminary experiments, 12.5 µM Triacsin C was selected for subsequent analyses. Zygotes were then allocated to the following experimental groups: Control, control + Triacsin C, delipidated, and delipidated + Triacsin C. To assess the time-dependent effect of Triacsin C, zygotes were cultured with 12.5 µM Triacsin C and monitored daily from 0.5*dpc* to 4.5*dpc* to determine the developmental window most sensitive to ACSL inhibition. This setup allowed the identification of the stage at which Triacsin C treatment could affect embryonic development.

A portion of zygotes, both delipidated and non-manipulated, was cultured in the presence of SBI-993, an inhibitor targeting ChREBP. SBI-993 was dissolved in DMSO, and initial concentrations of 12 and 15 µM were tested in control zygotes. Based on these preliminary experiments, the selected concentration was used to assess the effect in the following experimental groups: control, control treated with SBI-993, delipidated, and delipidated treated with SBI-993. Cleavage and developmental progression of treated embryos were analyzed relative to controls using three biological replicates, with approximately 30 embryos per group in each replicate.

### 2.5 RNA sequencing and differential expression analysis

Embryos were obtained from six hormonally stimulated female mice, yielding a total of approximately 70 embryos. These embryos were pooled and randomly allocated to either the delipidated or control (IVC) group (approximately 35 embryos per group). From each group, nine embryos were randomly selected for RNA-seq analysis and distributed into three independent samples, each consisting of three embryos per Eppendorf tube as experimental replicates. Embryo pools were transferred into 3.5 µL of sterile RNase-free PBS and stored at −80 °C until processing. Library preparations and sequencing reactions were conducted at GENEWIZ, LLC./Azenta US, Inc. (South Plainfield, NJ, USA). The SMARTer Stranded Total RNA-Seq Kit v2 - Pico Input Mammalian, 48 rxn (Takara Bio, Cat. ID: 634412) was used to prepare cDNA libraries with embryo samples processed directly without prior RNA extraction.

The protocol included ribosomal RNA depletion, strand-specific cDNA synthesis based on SMART technology, and library amplification according to the manufacturer’s instructions. The sequencing libraries were clustered onto a flow cell on an Illumina NovaSeq X plus instrument according to the manufacturer’s instructions. The samples were sequenced using a 2 × 150 paired-end (PE) configuration, targeting ∼10 M read-pairs per sample. Samples were sequenced with 30% Phi-X spike-in. The NovaSeq Control Software conducted image analysis and base calling. Raw sequence data (.bcl files) generated by the sequencer were converted into fastq files and de-multiplexed using Illumina’s bcl2fastq v.2.20 software. One mismatch was allowed for index sequence identification. After investigating the quality of the raw data, sequence reads were trimmed to remove possible adapter sequences and nucleotides with poor quality using Trimmomatic v.0.36. The trimmed reads were mapped to the *Mus musculus* reference genome available on ENSEMBL using the STAR aligner v.2.5.2b. BAM files were generated as a result of this step. Unique gene hit counts were calculated by using feature Counts from the Sub read package v.1.5.2. Only unique reads that fell within exon regions were counted. After extraction of gene hit counts, the gene hit counts table was used for downstream differential expression analysis. Using DESeq2, a comparison of gene expression between the groups of samples was performed. The Wald test was used to generate P values and Log2 fold changes. Genes with adjusted P values < 0.05 and absolute log2 fold changes >1 were called as differentially expressed genes for each comparison. RNA-seq gene expression data for all samples are listed in Supplementary Table S1.

### 2.6 Immunostaining

For immunofluorescence staining, the zona pellucida was removed using acid Tyrode’s solution (Piliszek et al., 2017; Czernik et al., 2022). Embryos were then transferred into 96-well plates pre-coated with 1% agar. Fixation was performed in 4% paraformaldehyde (PFA) in phosphate-buffered saline (PBS) containing 0.1% Tween-20% and 0.01% Triton X-100 for 20 min at room temperature. After fixation, embryos were permeabilised in 0.55% Triton X-100 in PBS for 20 min at RT. To quench free aldehyde groups remaining after fixation, embryos were incubated for 10 min at room temperature in 1 mL of ammonium chloride (NH_4_Cl) solution (0.0026 g/mL in PBS). Following this, embryos were washed three times in PBS, with each wash lasting 3 min. Non-specific binding was blocked by incubating embryos in 10% donkey serum in PBS for 40 min at room temperature. Primary antibodies were then applied at a 1:100 dilution in 10% donkey serum. The following antibodies were used: goat anti-SOX2 (Cat# AF2018, RRID:AB_355110, R&D Systems, USA), mouse anti-CDX2 (Cat# MU392A-UC, RRID:AB_3101998, BioGenex, USA), and rabbit anti-ChREBP Biotechne Cat# Nb400-135. Embryos were incubated with the primary antibody solution overnight (∼16 h) at 4 °C in the dark. After washing in PBS, secondary antibodies conjugated to Alexa Fluor dyes were applied at a 1:500 dilution in 10% donkey serum and incubated for 75 min at room temperature in the dark. During the final staining step, DNA was visualized using Hoechst 33342 (5 µg/mL) for 20–25 min at room temperature. Embryos were then transferred to glass-bottom dishes in a minimal volume of PBS and imaged using a NIS-Elements Confocal software (version 5.0, Nikon Instruments Inc.) and analyzed by Imaris software (version 9.8.2, Bitplane AG, Oxford Instruments) for advanced quantitative analysis and three-dimensional visualization.

### 2.7 Lipid droplet imaging and quantification

LDs were visualized using the fluorescent dye BODIPY 493/503 as previously described (Qiu and Simon, 2016). Stained samples were imaged using high-resolution confocal microscopy under identical acquisition settings across all developmental stages to ensure consistency for quantitative analysis. Confocal image stacks were processed and analyzed using Imaris software (version 9.8.2, Bitplane AG). Background noise was reduced using the Imaris *Background Subtraction* algorithm while preserving small LD signals. LDs larger than 0.01 µm^3^ were segmented using the Imaris *Surface* algorithm. Intensity thresholds were automatically determined from the BODIPY (488 nm) channel histogram, and a surface detail threshold of 0.1 µm^3^ was applied to eliminate noise and small artifacts. Misidentified objects, including dye aggregates, were manually corrected.

LDs were classified into size fractions (0.01–0.1, 0.1–0.5, 5–30, and >30 μm^3^), and their proportional distribution within the total LD population was quantified across developmental stages using part-of-whole analysis.

### 2.8 Statistical analysis

All experiments were conducted with a minimum of three independent biological replicates. Data were analyzed using GraphPad Prism (version 10; GraphPad Software, Inc., CA, USA). LD volume and LD number were analyzed using two-way ANOVA and One-way ANOVA to assess the effects of developmental stage and experimental group. For ChREBP foci number and intensity, nested t-tests and one-way ANOVA were used when multiple measurements were taken from the same biological replicate, and unpaired t-tests were applied for comparisons between independent groups. All statistical analyses were performed using GraphPad Prism. Statistical significance was considered as P < 0.05 (*), P < 0.001 (***), and P < 0.0001 (****).

### 2.9 Reagents

**Table:** 

Reagent	Source	Catalog No.
Animal model		
C57Bl/10 × CBA/H	IGAB PAN	​
C57Bl/6	Jackson laboratory	000664
CBA/Ca	Jackson laboratory	000654
Hormones		
Pregnant mare serum gonadotropin (PMSG)	Bio vendor R&D	Cat# RP17827225000
hCG	MSD animal health	Cat# 018533
Embryo culture reagents		
Free fatty acid BSA	Sigma-Aldrich	CAS-No: 9048-46-8
Dimethyl sulfoxide (DMSO)	Sigma-Aldrich	Cat# D2650
Mineral oil	Sigma-Aldrich	Cat# M8410
Enzymes and treatment reagents		
Triacsin C	MCE, MedChem express	Cat# HY-N6707
SBI-993	MCE, MedChem express	Cat. No.: HY-122682
Tyrode′s solution, Acidic	Sigma-Aldrich	Cat# T1788
Cytochalasin B	R&D system	Cat# C2743
Sucrose (C_12_H_22_O_11_)	Sigma-Aldrich	Cat# 59738
Hyaluronidase	Sigma-Aldrich	Cat# H-3506
siRNA-ChREBP	Euro genetics	SR-NP001-001
Staining reagents		
BODIPY 493/503	Thermo Fisher scientific	Cat# D3922
Hoechst stain solution	Sigma-Aldrich	Cat# H6024
Chemical reagents		
Agar	Sigma-Aldrich	Cat# A1296
Paraformaldehyde (PFA)	Sigma-Aldrich	Cat# 47608
Phosphate-buffered saline (PBS)	Roth	Cat# 1112.2
Tween 20	Sigma-Aldrich	Cat# P9416
Triton X-100	Sigma-Aldrich	Cat# T9284
Ammonium chloride (NH_4_Cl)	Sigma-Aldrich	Cat# AS434
Antibodies		
ChREBP	Biotechne	Cat# Nb400-135
Anti calnexin	Abcam	Cat# Ab112995-100
Anti-CDX2	Biogenex, USA	Cat# MU392A-UC
Anti-SOX2	Bio-techne	Cat# AF2018
Donkey anti-goat IgG (H+L) cross-adsorbed secondary antibody, alexa Fluor™ 647	Thermo Fisher scientific	Cat# A21447
Donkey anti-rabbit IgG (H+L) highly cross-adsorbed secondary antibody, Alexa Fluor™ 568	Thermo Fisher scientific	Cat# A10042
Software and algorithms		
Graphpad prism version 10	GraphPad	RRID:SCR_002798
Imaris microscopy image analysis 9.8.2 software	Oxford instruments	RRID:SCR_007370
NIS-elements confocal	Nikon instruments Inc.	RRID: SCR_014329

## 3 Results

### 3.1 The removal of lipids via delipidation and its effect on mouse pre-implantation embryos development

**FIGURE 1:**
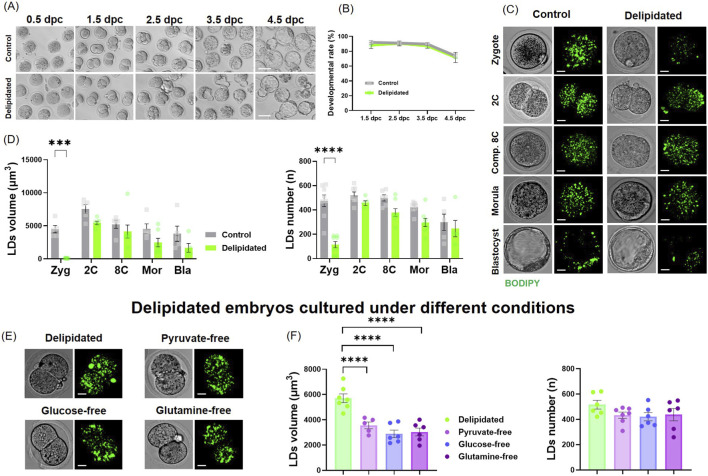
Pre-implantation development of delipidated zygotes. **(A)** Embryos derived from delipidated and non-delipidated embryos cultured in free–fatty-acid–free KSOM medium from 0.5*dpc* to 4.5*dpc*. **(B)** Percentage of embryos reaching each developmental stage across 0.5–4.5*dpc* in delipidated versus non-delipidated groups. Data from three biological replicates were analyzed using two-way ANOVA. **(C)** LDs (BODIPY 493/503, green) from zygote to blastocyst stages. **(D)** Quantification of total LD volume and total LD number per embryo. Data from three biological replicates (n = 32 embryos) were analyzed using one-way ANOVA, **** indicates p < 0.0001). **(E)** LDs (BODIPY 493/503, green) in delipidated embryos cultured in KSOM medium lacking pyruvate, glucose, and glutamine. **(F)** Total LD volume and number in delipidated two-cell embryos under nutrient-deprived conditions were quantified. Data from three biological replicates (n = 32 embryos) and were analyzed using ordinary one-way ANOVA with (p < 0.0001). Scale bars: 65 μm in **(A)** and 10 μm in **(C,E)**.

After complete removal of lipids from zygote-stage embryos, the embryos were cultured in fatty acid–free KSOM and monitored for development from 0.5*dpc* to 4.5*dpc* (Figure 1A). Control and delipidated embryos developed normally with the same efficiency, showing no significant differences in cleavage, compaction, or blastocyst formation (Figure 1B). We tracked LDs using BODIPY staining from the zygote to the blastocyst stage (Figure 1C). As expected, LDs were absent immediately after removal at the zygote stage; however, no significant difference in LD volume and numbers was observed between the control and delipidated groups. These differences persisted despite embryos being cultured in fatty acid–free KSOM (Figure 1D; Supplementary Figure S1). Following delipidation, embryos were cultured without pyruvate, glucose, and glutamine. Although LDs regenerated under all nutrient-depleted conditions, a significant reduction in LD volume was observed in the absence of pyruvate (****, *p* < 0.0001) as well as in the absence of glucose and glutamine (****, *p* < 0.0001) (Figures 1E,F; Supplementary Figure S1).

### 3.2 Inhibition of *de novo* lipogenesis (DNL) through ACSL activity arrests preimplantation development

**FIGURE 2:**
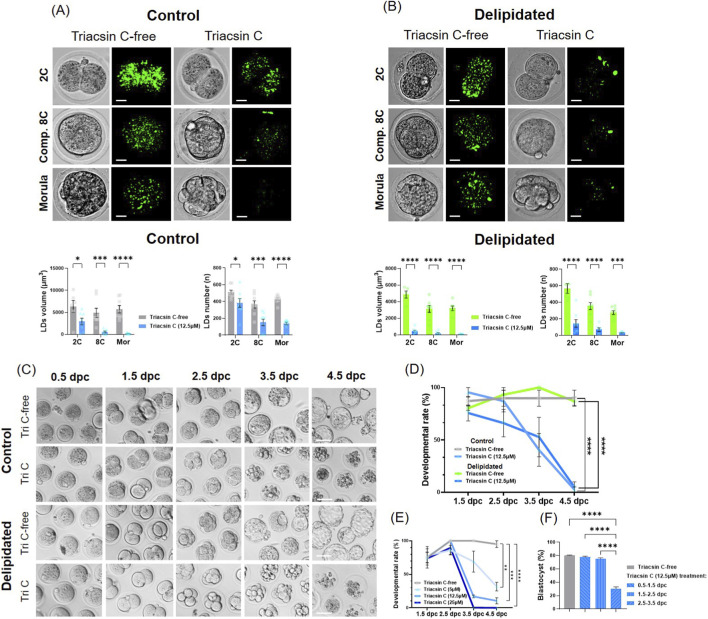
ACSLs’ inhibition by Triacsin affects LD accumulation and embryo development. **(A,B)** Up: LDs (BODIPY 493/503, green) among the four groups from the 2-cell stage to the morula stage. Images are representative of F1 embryos (at least n = 10 embryos per groups). Down: Quantification of total LD volume and total LD number per embryo. Data in three biological replicates were analyzed using two-way ANOVA (*p < 0.05; ***p < 0.001; treatment effect: F (1,40) = 47.08 ****p < 0.0001 (scale bars: 10 μm). **(C)** Representative images of embryos from four experimental groups with quantification of developmental progression. **(D,E)** Data show the percentage of embryos reaching each developmental stage for all four groups. Data in three biological replicates (n = 20 embryos per group) were analyzed using two-way ANOVA (*p < 0.05; ***p < 0.001; ****p < 0.0001). **(F)** The graph represents the blastocyst development rate among the various time-dependent Triacsin C treatments. A statistically significant difference was detected only in the Day 3 group. The data were analyzed using ordinary one-way ANOVA (****p < 0.0001). Scale bars: 10 μm in **(A)** and 65 μm in **(C)**.

As expected, Triacsin C, an inhibitor of Acyl-CoA synthetases (ACSLs), blocked DNL, thereby impairing lipid droplet accumulation in both control and delipidated embryos (Figures 2A,B; Supplementary Figure S2). Consistent with previous reports (Aizawa et al., 2019), zygotes cultured in the presence of Triacsin C exhibited arrest of preimplantation development in both IVC control and delipidated embryos (Figures 2C,D). Dose–dependent response analysis using increasing concentrations of Triacsin C (5, 10, and 25 µM) identified the morula stage as particularly sensitive to ACSL inhibition, with developmental defects evident even at lower concentrations. Notably, the transition from morula to blastocyst was strongly impaired at higher doses of the inhibitor (12.5 and 25 µM), as shown by the concentration–response curves (Figure 2E). To dissect the temporal requirement for ACSL activity, Triacsin C treatment was restricted to 24-h windows at different stages of preimplantation development (Figure 2F): 0.5–1.5*dpc*zygote to 2-cell stage, first day of IVC), 1.5–2.5*dpc* (2-cell to 4–8-cell stage, second day of IVC), and 2.5–3.5*dpc* (morula to blastocyst stage, third day of culture). A significant reduction in blastocyst formation was observed only when ACSL inhibition was applied at 2.5*dpc*, highlighting the morula-to-blastocyst transition as a critical window for ACSL-dependent lipid metabolism during preimplantation embryonic development.

### 3.3 ChREBP drives lipid droplet regeneration

**FIGURE 3:**
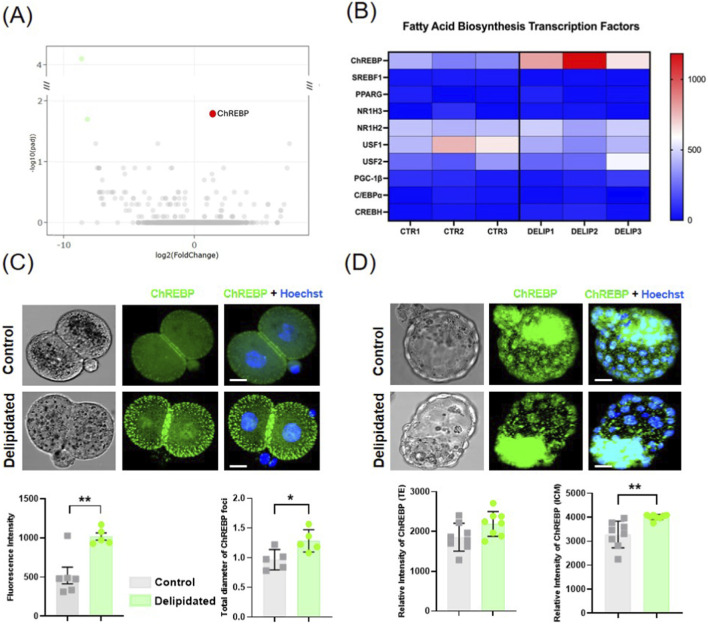
ChREBP during early embryo development. **(A)** The global transcriptional change across the groups compared was visualized by a volcano plot. Each data point in the scatter plot represents a gene. The log2 fold change of each gene is represented on the x-axis and the log10 of its adjusted p-value is on the y-axis. Genes with an adjusted p-value less than 0.05 and a log2 fold change greater than 1 are indicated by red dots. These represent upregulated genes. Genes with an adjusted p-value less than 0.05 and a log2 fold change less than −1 are indicated by green dots. These represent downregulated genes. **(B)** Heatmap was used to visualize the expression profile of the top 10 transcription factors related to lipid metabolism. **(C)** Immunofluorescence images of ChREBP in the 2-cell. Histograms: quantification of ChREBP foci diameter and nuclear fluorescence intensity were analyzed using a nested t-test (*p = 0.0272, **p = 0.0040). **(D)** Immunofluorescence images of ChREBP in the blastocysts. Histograms: relative intensity of Trophectoderm (TE) and Inner Cell Mass (ICM) was analyzed using the nested t-test (**p < 0.0043). Scale bars: 10 μm in **(C)** and 20 μm in **(D)**.

To investigate the molecular mechanisms underlying the regeneration of newly formed LDs after delipidation, RNA sequencing was performed on 2-cell stage embryos 24 h post zygote delipidation in respect to the controls. RNA sequencing analysis revealed that the transcription factor carbohydrate response element-binding protein (ChREBPα) was the unique significant mRNA upregulated in delipidated embryos compared to IVC control embryos (Figures 3A,B). Differentially expressed genes between control and treated groups are listed in Supplementary Table S1. However, we performed Gene Ontology (GO) enrichment analysis on the 2,000 genes with the highest positive log fold change (Log_2_FC). In the treatment condition, the analysis suggested a trend toward enrichment of mitochondrial-related processes, including mitochondrial gene expression, translation, and organization (Supplementary Figure S3). Although this enrichment did not reach statistical significance, the observed trend may indicate a reactivation of embryonic metabolism in delipidated embryos, consistent with the metabolic recovery associated with lipid droplet rescue. At the protein level, ChREBP appeared expressed as distinct green foci in control 2-cell stage embryos, primarily localized at the cellular cortex beneath the plasma membrane. This is consistent with previous reports of non-nuclear localization of ChREBP (Noordeen et al., 2012), suggesting that ChREBP was predominantly in its inactive form. In contrast, delipidated 2-cell stage embryos exhibited larger and more abundant ChREBP-positive signals in the cytoplasm and subtle nuclear signal. In addition, the ChREBP fluorescence intensity was significantly higher in delipidated compared to different controls (Supplementary Figure S4). At the blastocyst stage, ChREBP was expressed in all cells, although in the TE it was almost exclusively restricted to the nucleus. Delipidation led to higher fluorescence intensity in the inner cell mass (ICM), with partial nuclear localization, indicating ChREBP activation in response to LD depletion. (Figures 3C,D). No significant change in ChREBP localization was observed in the trophectoderm (TE), highlighting a lineage-specific response to LD depletion. This suggests that ChREBP may be partially active in regulating lipid metabolism at the blastocyst stage (Figure 3D). Overall, delipidation leads to increased ChREBP expression and enhanced nuclear localization, highlighting its role as a key regulator of metabolic adaptation to LD deprivation in early embryos.

### 3.4 ChREBP inhibition blocks lipid droplet regeneration and preimplantation embryo development

**FIGURE 4:**
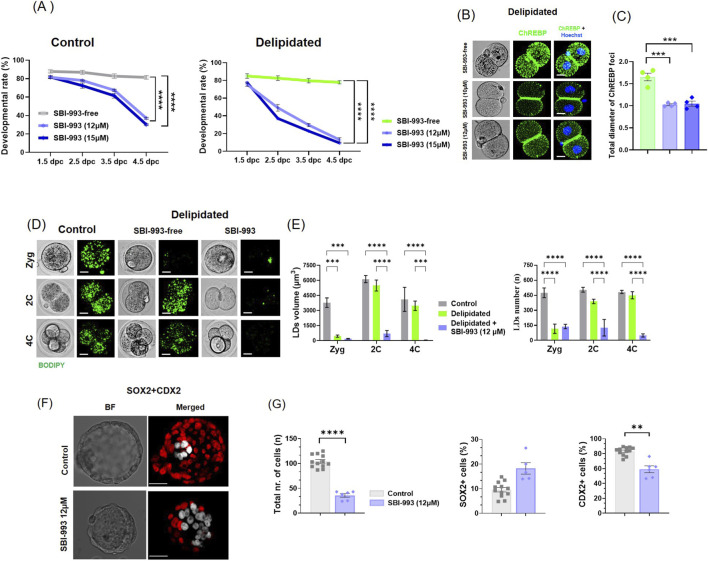
Inhibition of ChREBP. **(A)** Representative images showing the effect of different concentrations of the ChREBP inhibitor SBI-993 on embryo development in control and delipidated embryos. Embryos were treated with SBI-993 at various concentrations and cultured from 0.5*dpc* to 4.5*dpc*. Percentage of embryos reaching each developmental stage across 0.5–4.5*dpc* in control and delipidated embryos versus ChREBP inhibitor SBI-993 groups. Data represent three control and treated biological replicates (F1 embryos, n = 60 from 5 females) and were analyzed using two-way ANOVA (treatment effect: F _(2,24)_ = 246.7, ****p < 0.0001) and three delipidated and treated biological replicates (F1 embryos, n = 60 from 5 females) and were analyzed using two-way ANOVA (treatment effect: F _(2, 24)_ = 1,540, ****p < 0.0001). **(B)** Immunofluorescence images of ChREBP in the 2-cell in delipidated embryos treated with SBI-993 at 10 μM and 12 µM. **(C)** Quantification of ChREBP foci size was significantly different (***p < 0.001, nested one-way ANOVA). The data represent three biological replicates. **(D)** Representative confocal images of lipid droplets (LDs; BODIPY 493/503, green) from three groups: control, delipidated, and delipidated treated with SBI-993, from the 2-cell to the 4-cell stage. **(E)** Quantification of total LDs volume and total LDs number per embryo. Datasets represent three biological replicates. Images are representative of F1 embryos (n = 90) collected from 8 mice and were analyzed using two-way ANOVA (***p < 0.001; treatment factor: F _(2, 42)_ = 40.28 ****p < 0.0001). **(F)** Representative confocal scans of 4.5*dpc* control and SBI-933–treated embryos stained for SOX2 (white) and CDX2 (red). **(G)** The numbers of CDX2- and SOX2-positive cells were quantified, and statistical significance was assessed using a t-test (**p < 0.01; **p < 0.0001). Scale bars: 10 μm in **(B,D)**, 20 μm in **(F)**.

To investigate the role of ChREBP during preimplantation development, embryos were treated with the ChREBP inhibitor SBI-993 (Benichou et al., 2024). Treatment with SBI-993 (12–15 µM) resulted in a significant impairment of embryonic development between 3.5 and 4.5*dpc* compared with their respective untreated controls, thus resulting in a developmental block at the morula-to-blastocyst transition stage (Figure 4A). This effect was associated with a lack of the observed increase in ChREBP foci diameter at the 2-cell stage (Figures 4B,C) and was accompanied by a complete blockade of LD regeneration during the early stages of embryonic development (Figures 4D,E; Supplementary Figure S5). Moreover, we analyzed blastocyst lineage-specific markers SOX2 and CDX2 to stain the epiblast and the trophectoderm, respectively. No differences were observed comparing delipidated with control embryos in the total number of cells, nor in the number of SOX2- and CDX2-positive nuclei (Supplementary Figure S6). However, when control embryos were treated with SBI-993, there was a significant reduction of the total number of cells, which was mediated solely by a severe decrease in CDX2-positive TE cells (Figures 4F,G). Together, these observations demonstrate that ChREBP is required for LD biogenesis in response to lipid depletion and that successful preimplantation embryo development is highly dependent on ChREBP activity.

### 3.5 Co-localization of cytoplasmic ChREBP with LD and endoplasmic reticulum in early embryos

**FIGURE 5:**
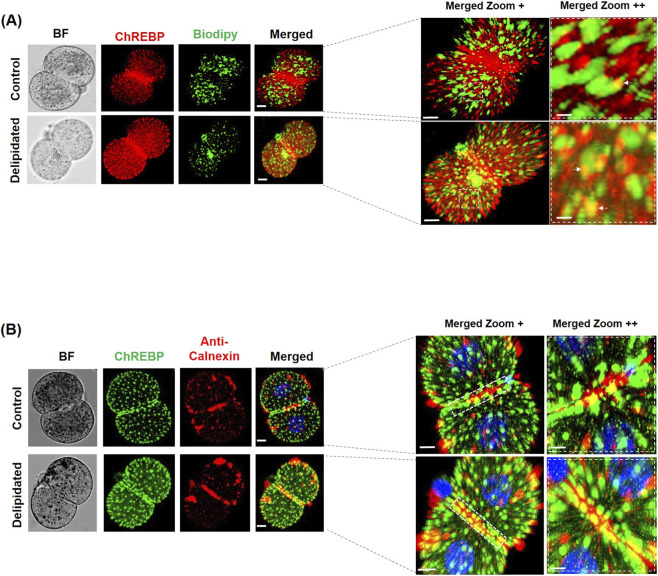
Citoplasmic ChREBP localization. **(A)** Representative confocal images showing the localization of ChREBP and lipid droplets (LDs; BODIPY 493/503, green and ChREBP in red) at the 2-cell stage. ChREBP and LD signals were observed mainly as separate signals, with a small portion showing co-localization (examples indicated by white arrows). Co-localization events were more evident in embryos derived from delipidated zygotes. **(B)** Representative confocal images showing the localization of ChREBP and the endoplasmic reticulum marker Calnexin in control and delipidated 2-cell embryos. ChREBP shows a peripheral and cortical distribution in control embryos, partially overlapping with Calnexin-positive structures. In embryos derived from delipidated zygotes, ChREBP signal appears increased and more broadly distributed, with enhanced proximity to Calnexin-labeled regions. Enlarged views highlight representative areas (dashed boxes). Scale bars: 10 μm in left panels, 5 μm in zoom+, and 3 μm in zoom++.

Immunofluorescence analysis revealed a distinct cytoplasmic localization of ChREBP in early mouse embryos. At the 2-cell stage, ChREBP in control embryos appeared as discrete punctate signals predominantly localized at the cell cortex and beneath the plasma membrane, with limited overlap with LDs labeled by BODIPY (Figure 5A). This spatial distribution indicates that, under physiological conditions, ChREBP is largely retained in the cytoplasm and likely maintained in an inactive state. In contrast, embryos derived from delipidated zygotes exhibited a marked increase in ChREBP signal intensity, accompanied by a broader cytoplasmic distribution and enhanced spatial proximity to LDs, as highlighted in magnified views (Figure 5A, dashed squares). To further define the subcellular localization of cytoplasmic ChREBP, co-immunostaining with the endoplasmic reticulum (ER) marker Calnexin was performed (Figure 5B). In control embryos, ChREBP partially associated with Calnexin-positive structures at the cell periphery (Figure 5B, dashed box). Following delipidation, ChREBP displayed a stronger and more diffuse cytoplasmic signal with increased proximity to ER-enriched regions (Figure 5B, dashed box). Together, these observations suggest a previously unrecognized role for cytoplasmic ChREBP in LD biogenesis originating from the endoplasmic reticulum during early embryonic development.

## 4 Discussion

Our findings demonstrate that nutrient sensing and energy allocation constitute a central feature of early embryonic metabolism and are essential for survival. Here, we describe an adaptive metabolic program mediated by ChREBP that safeguards development under conditions of lipid deficiency. Early embryos exhibit active DNL and store neutral lipids as triglycerides within LDs (Kong and Gao, 2024; Zhang et al., 2024). Notably, as previously demonstrated by Aizawa et al. (2019), acute removal of LDs from embryos results in their rapid reconstitution, without immediate detrimental effects on embryonic development.

This phenomenon has complicated the functional role of LDs in early embryonic survival. We show that mechanical delipidation of the zygote triggers, in parallel with LD recovery, a marked upregulation of ChREBP (*Mlxipl*) mRNA and protein expression, which emerges as the principal differentially expressed gene. ChREBPα is a transcription factor containing both nuclear import and export signals, as well as a DNA-binding domain, and it coordinates the expression of enzymes that channel glycolytic intermediates into lipid biosynthesis (Uyeda and Repa, 2006; Nakagawa et al., 2013). In metabolically specialized tissues such as the liver, nuclear abundance of transcriptionally competent ChREBP is high under carbohydrate-rich conditions and reduced under a high-fat diet (Sato et al., 2016). Consistently, in our study, ChREBP expression was higher under fatty acid-free conditions, and following lipid reserves removal. ChREBP protein localized predominantly to cytoplasmic foci at the interfaces of intracellular membranes and the cytoplasm in 2-cell control embryos, which resulted significantly enlarged after LD removal by delipidation. This localization pattern suggests that ChREBP is maintained in an inactive state, potentially reflecting a phosphorylated, transcriptionally inactive pool during early cleavage stages.

Accordingly, expression of ChREBP target genes remained low after delipidation, indicating that its transcriptional activity is temporally regulated (Iizuka and Horikawa, 2008; Sakiyama et al., 2008; Davies et al., 2008). Functional activation of ChREBP likely occurs at the transition from morula to blastocyst stage, when we found the most abundant nuclear ChREBP expression in ICM and TE cells, as compared to cleavage stages, when ChREBP was expressed mainly in peri-membrane foci. This developmental window is characterized by increased glucose uptake, enhanced flux through the pentose phosphate pathway (PPP), and nuclear translocation of ChREBP (Davies et al., 2008; Leese, 2012). This is consistent with the production of xylulose-5-phosphate, a known activator of ChREBP dephosphorylation and nuclear import (Kabashima et al., 2003; Dentin et al., 2005). Consequently, embryos may initiate a ChREBP-dependent transcriptional program in response to lipid depletion, although its full functional execution is delayed until metabolic conditions become permissive at the blastocyst stage. Notably, early cleavage-stage embryos are generally considered glycolytically quiescent and largely glucose-independent due to limited glucose transporter expression (Hogan et al., 1991; Morita et al., 1994).

Our data suggest that the metabolic state may be reversible under conditions of reduced LD storage. Indeed, LD regeneration in delipidated embryos partially depended on the availability of glucose and glutamine in the culture medium, indicating unexpected metabolic flexibility during early development. Consistent with previous reports, inhibition of DNL by Triacsin C resulted in LD loss and development arrest at the morula stage (Aizawa et al., 2019; Valente et al., 2019), identifying this transition as particularly sensitive to disruptions of embryonic homeostasis, compromising the morula-to-blastocyst transition and embryonic survival. Furthermore, inhibition of ChREBP by SBI-993 impaired blastocyst development in control, not-delipidated embryos, thus indicating its important role in embryonic LD dynamics and survival to the blastocyst stage. Concurrently, inhibition of ChREBP in delipidated embryos causes developmental arrest at an earlier stage, supporting the view that ChREBP upregulation represents an adaptive metabolic response to lipid deprivation and is critical for developmental progression. The mechanism by which ChREBP senses lipid stores remains intriguing and still unknown. We propose that the flux through fatty acid activation, specifically acyl-CoA formation rather than the physical presence of LDs, *per se* regulates ChREBP expression and activity. Our data suggest that feedback regulation of ChREBP transcription could depend on the Acyl-CoA/free fatty acid (FFA) ratio, with increased ratios promoting ChREBP expression and reduced ratios suppressing it. This interpretation is supported by the evidence that lipid-rich reduce ChREBP mRNA in mouse liver (Dentin et al., 2005; Jois et al., 2016). Beyond their role as an energy reservoir, LDs are increasingly recognized as dynamic organelles with regulatory functions in embryonic development. LDs contribute to early lineage specification in the blastocyst, reinforcing the concept of metabolic programming during early development. Consistent with previous studies (Watanabe et al., 2010; Kajdasz et al., 2020), the ICM retains abundant LDs, whereas the TE contains markedly fewer (Figure 1C), reflecting fundamental metabolic divergence between these lineages, although this lineage divergence could vary in different mouse strains and mammalian species. The ICM must accumulate LDs to support epiblast development and subsequent peri-implantation events, such as gastrulation (Mau et al., 2022); in contrast, the TE is more metabolically flexible, relying on available energy sources to meet the high demands of the morula-to-blastocyst transition. LDs are therefore essential for driving the morula-to-blastocyst transition, a process that requires enhanced fatty-acid β-oxidation to generate sufficient ATP to fuel blastocoel formation and Na^+^ pumping (Dunning et al., 2010). Accordingly, we show that ChREBP inhibition arrests blastocyst formation by impairing CDX2 expression and trophectoderm (TE) development, while not affecting SOX2-positive cells. While LDs regenerate during early cleavage stages after delipidation, ChREBP remains mainly cytoplasmic; in contrast, at the blastocyst stage, ChREBP predominantly translocates to the nucleus, activating a transcriptional program that redirects glycolytic flux toward lipid biosynthesis and influences blastocyst differentiation.

Interestingly, recent studies have reported cytoplasmic accumulation of ChREBP in discrete foci (Davies et al., 2008; Ortega-Prieto and Postic, 2019) and our results show that this phenomenon also occurs in preimplantation cleaving embryos. These results indicate that ChREBP can associate with membrane compartments of LDs in an inactive, phosphorylated state, poised for activation upon metabolic stimulation (Mejhert et al., 2020). In our experiments, ChREBP foci were primarily localized at the cell cortex and distinct from LDs, although a co-localization was observed in embryos undergoing active LD regeneration following delipidation. Synchronously, ChREBP foci in delipidated embryos co-localized in more proximity to endoplasmic reticulum-enriched regions, from which LDs originate (Beller et al., 2010; Welte, 2015; Salo et al., 2016), and with LD particularly in delipidated embryos. These findings suggest a previously unrecognized cytoplasmic role for ChREBP in lipid droplet biogenesis from the ER, opening new avenues for investigation.

In summary, our findings establish ChREBP as a central regulator of lipid droplet synthesis and embryonic survival during preimplantation stages. Through this pathway, ChREBP actively governs cellular energy balance, dynamic lipid remodeling, and morphogenetic progression to the blastocyst stage. Elucidating how ChREBP integrates lipid storage with metabolic flux and developmental timing reveals a fundamental mechanism underlying metabolic control of early development and energy homeostasis. Further investigation of ChREBP depletion via Trim-Away or genetic approaches using homozygous ChREBP knockout oocytes will further elucidate the role of ChREBPα in lipid droplet biogenesis during preimplantation development.

## Data Availability

The datasets presented in this study have been deposited in the Gene Expression Omnibus (GEO) under accession number GSE326820.
