# Supplementary material for: ChREBPα: a central metabolic sensor driving lipid droplet renewal in preimplantation mouse embryos
Source: Front Cell Dev Biol. 2026 Apr 23;14:1793255. doi: 10.3389/fcell.2026.1793255 (PMC13149441; doi:10.3389/fcell.2026.1793255)
Supplement: Supplementary file 1 [file DataSheet1.pdf]

Supplementary Material

1.1 Supplementary Figures

(S1)

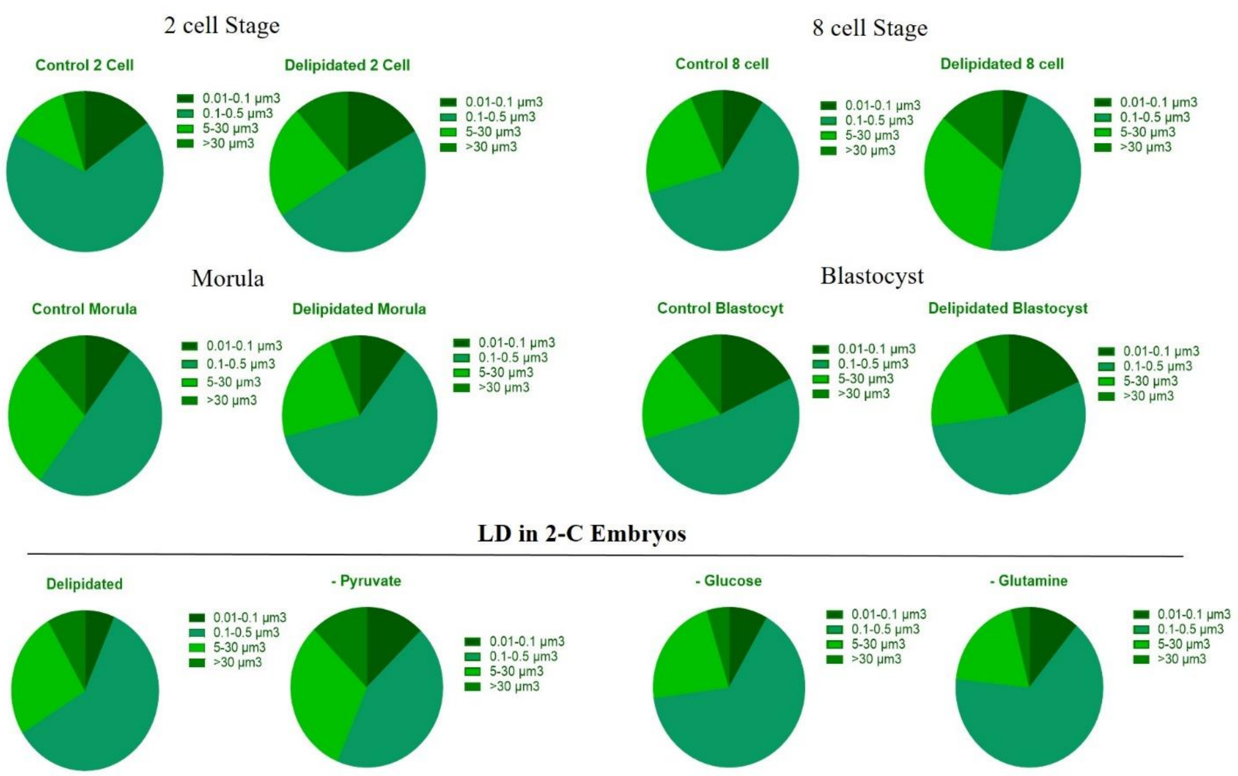

Supplementary Figure 1. Quantification of LD size in ~~Delipidated~~ embryos. Quantification of LD size fractions (0.01–0.1, 0.1–0.5, 5–30, and > 30 μm³) and their proportional distribution within the total LD population across developmental stages ~~in delipidated and control embryos, and in~~ 2-cell stage delipidated embryos cultured under different conditions.

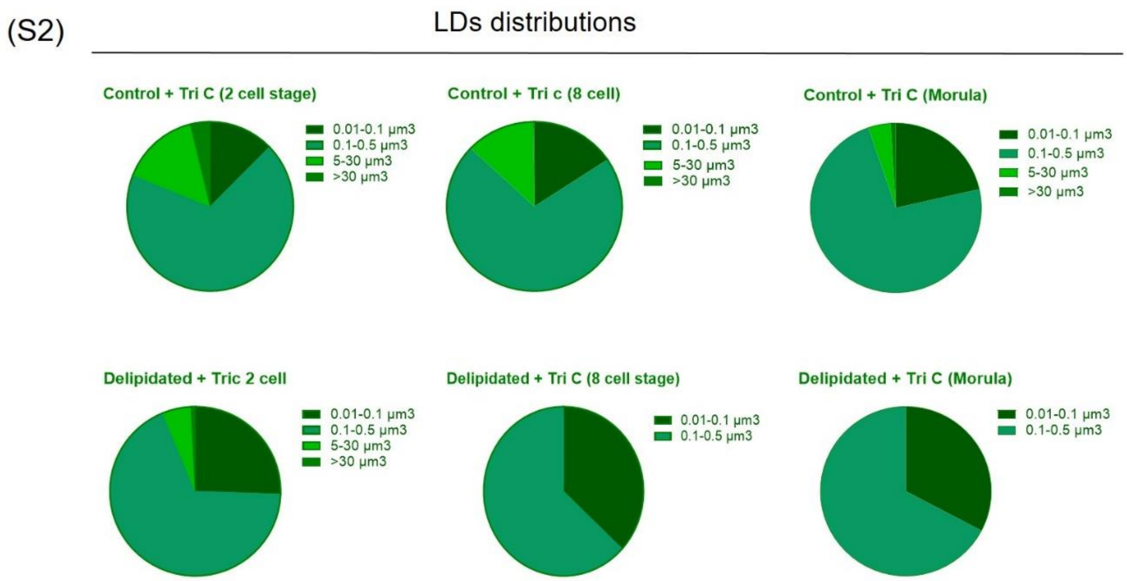

Supplementary figure 2. Distribution of LDs across different size fractions (0.01–0.1, 0.1–0.5, 5–30, and >30 µm³), expressed as a proportion of the total LD population in control and delipidated embryos treated with Triacsin-C.

(S3)

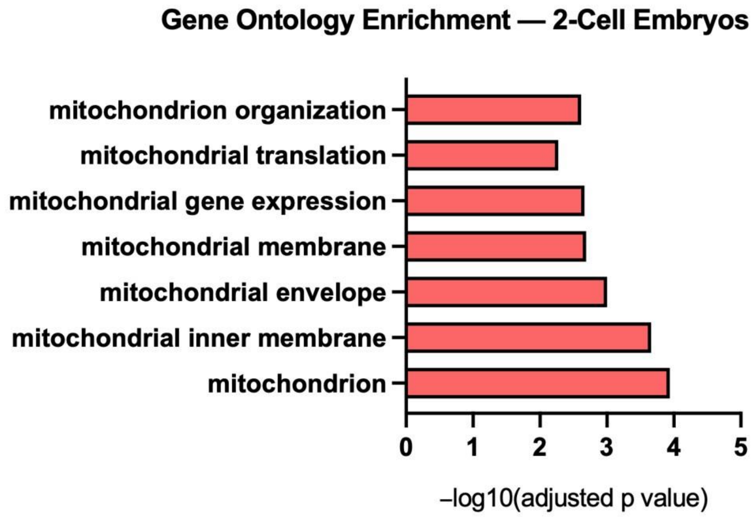

Supplementary Figure 3. Gene Ontology (GO) enrichment analysis on the 2,000 genes with the highest positive log fold change (LogFC) in 2-Cell embryos from delipidated vs control zygotes.

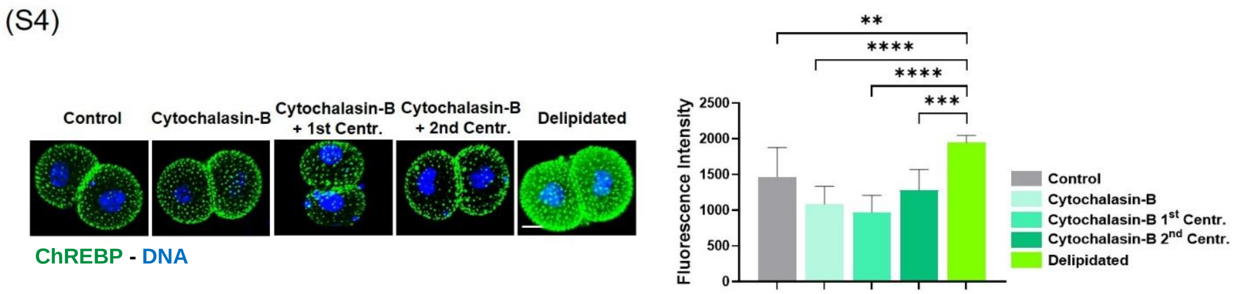

Supplementary figure 4. Representative immunofluorescence images of ChREBP in delipidated embryos compared with different control groups at the 2-cell stage. ChREBP fluorescence intensity were quantified and analyzed using one-way ANOVA (**$p < 0.01$, ***$p < 0.001$, ****$p < 0.0001$). Scale bar: 10μm

(S5)

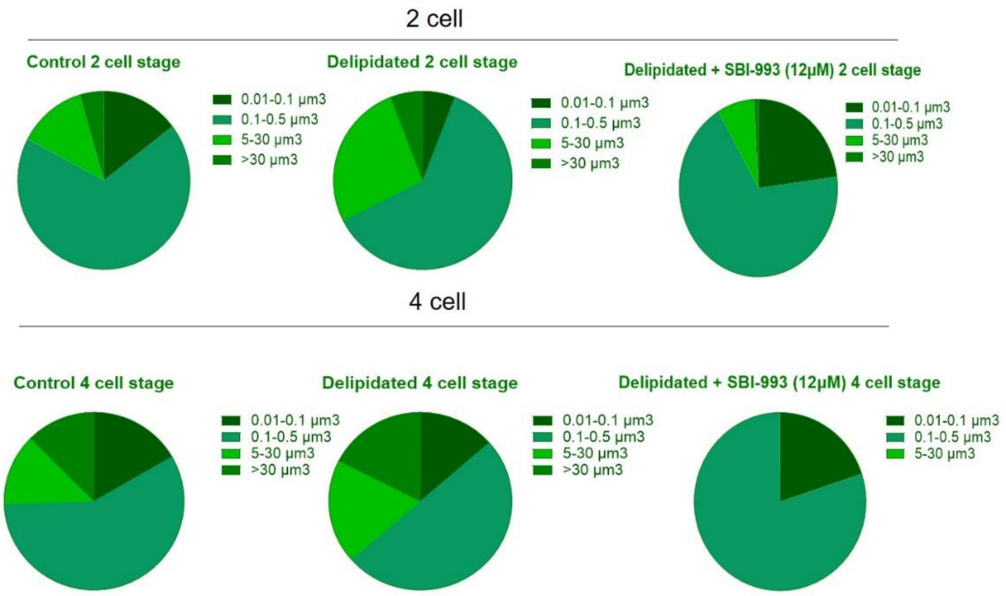

Supplementary figure 5. Distribution of LDs across different size fractions (0.01–0.1, 0.1–0.5, 5–30, and >30 µm^3), expressed as a proportion of the total LD population in control and delipidated embryos with or without SBI-993.

(S6)

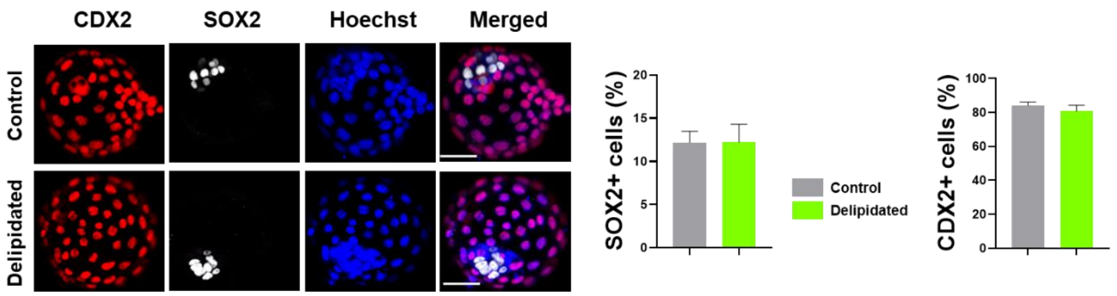

Supplementary figure 6. Lineage specification was evaluated by quantifying the number of CDX2- and SOX2-positive cells in control and delipidated blastocysts. Statistical analysis using an unpaired t-test revealed no significant differences between the groups. Scale bar: 20μm.
